# A Unique Regulator Contributes to Quorum Sensing and Virulence in *Burkholderia cenocepacia*

**DOI:** 10.1371/journal.pone.0037611

**Published:** 2012-05-18

**Authors:** Eoin P. O'Grady, Duber F. Viteri, Pamela A. Sokol

**Affiliations:** Department of Microbiology, Immunology and Infectious Diseases, University of Calgary, Calgary, Alberta, Canada; University of Malaya, Malaysia

## Abstract

*Burkholderia cenocepacia* causes chronic and life-threatening respiratory infections in immunocompromized people. The *B. cenocepacia N*-acyl-homoserine lactone (AHL)-dependent quorum sensing system relies on the production of AHLs by the synthases CepI and CciI while CepR, CciR and CepR2 control expression of many genes important for pathogenesis. Downstream from, and co-transcribed with *cepI*, lies BCAM1871 encoding a hypothetical protein that was uncharacterized prior to this study. Orthologs of *B. cenocepacia* BCAM1871 are uniquely found in *Burkholderia* spp and are conserved in their genomic locations in pathogenic *Burkholderia*. We observed significant effects on AHL activity upon mutation or overexpression of BCAM1871, although these effects were more subtle than those observed for CepI indicating BCAM1871 acts as an enhancer of AHL activity. Transcription of *cepI*, *cepR* and *cciIR* was significantly reduced in the BCAM1871 mutant. Swimming and swarming motilities as well as transcription of *fliC*, encoding flagellin, were significantly reduced in the BCAM1871 mutant. Protease activity and transcription of *zmpA* and *zmpB*, encoding extracellular zinc metalloproteases, were undetectable in the BCAM1871 mutant indicating a more significant effect of mutating BCAM1871 than *cepI*. Exogenous addition of OHL restored *cepI*, *cepR* and *fliC* transcription but had no effect on motility, protease activity or *zmpA* or *zmpB* transcription suggesting AHL-independent effects. The BCAM1871 mutant exhibited significantly reduced virulence in rat chronic respiratory and nematode infection models. Gene expression and phenotypic assays as well as vertebrate and invertebrate infection models showed that BCAM1871 significantly contributes to pathogenesis in *B. cenocepacia*.

## Introduction

Diverse pathogenic bacteria often utilize cell-cell communication (quorum sensing, QS) systems to communicate with neighboring cells. These QS systems enable organisms to produce and perceive chemical signals resulting in altered gene expression and phenotypes contributing to pathogenesis. In Gram-negative bacteria, the most widely-studied QS system involves production of *N*-acyl-homoserine lactones (AHLs) by synthases of the LuxI protein family (for review see [1], [2] . During bacterial growth these AHLs are released from bacterial cells into the surrounding medium. As cell density increases and the AHL concentrations become relatively high, sufficient numbers of these molecules are found inside bacterial cells where they reversibly bind cognate transcriptional regulators that are members of the LuxR protein family. AHL binding induces a conformational change activating the transcriptional regulator. Transcriptional activation or repression can be exerted by LuxR proteins in the AHL-bound or unbound state. Additionally, there are LuxR homologs that are not genetically linked to an AHL synthase gene and these are called orphan or solo LuxR homologs [3].

The *Burkholderia cepacia* complex (Bcc) is a group of 17 closely-related bacteria that are commonly found in soil, water and agricultural environments and have importance in medicine, agriculture and biotechnology [4], [5]. Bcc cause nosocomial infections as well as severe illnesses in neonates, patients with cystic fibrosis (CF) or cancer, transplant recipients and other immunocompromized patients [6]. *B. cenocepacia* is transmissible, intrinsically resistant to antibiotics and contributes to lung and bloodstream infections resulting in significantly decreased survival rates in CF and chronic granulomatous disease patients [7], [8]. Some CF patients succumb to “cepacia syndrome”, a rapid deterioration in lung function associated with necrotizing pneumonia, bacteremia and sepsis [8], [9]. Bloodstream dissemination of *B. cenocepacia* can also occur in individuals receiving hemodialysis [10].

The *B. cenocepacia* AHL-dependent QS system is composed of two AHL synthases and three regulatory proteins enabling fine-tuned regulation of gene expression [11], [12], [13]. The CepIR system is present in all species of the Bcc [11], [14], [15], [16] while the CciIR system is only present in *B. cenocepacia* containing the *c*eno*c*epacia *i*sland (cci) found in highly transmissible strains [15]. All *B. cenocepacia* strains contain the orphan LuxR homolog CepR2 [13]. CepI is primarily responsible for the synthesis of *N*-octanoyl-L-homoserine lactone (OHL) and minor amounts of *N*-hexanoyl-L-homoserine lactone (HHL) [11]. CciI primarily synthesizes HHL with lesser amounts of OHL produced [12]. The functional significance of the *B. cenocepacia* AHL-mediated QS system is underscored by the fact that AHL activity is maintained in the majority of sputum isolates recovered from chronically-infected CF patients [17].

There is a complex regulatory interrelationship between the *B. cenocepacia* QS systems. Transcription of *cepI* and the *cciIR* operon depends on CepR, but CciR negatively regulates the expression of *cepI* and the *cciIR* operon thus allowing regulatory feedback [12], [16]. Furthermore CepR, CciR and CepR2 are negative autoregulators [12], [13], [16]. Additionally, CepR activity can be inhibited by excess amounts of HHL which is the primary product of *cciI* [18]. For the most part, CepR is a positive regulator while CciR is a negative regulator of gene expression, with the vast majority of commonly-regulated genes being reciprocally regulated [19]. CepR2 positively or negatively influences gene expression and can influence gene expression in the absence of AHLs [13].

The *B. cenocepacia* global regulators CepIR and CciIR control protease, lipase and chitinase activities, swimming and swarming motilities, siderophore production, biofilm formation and maturation, as well as expression of genes encoding components of efflux pumps, flp type pili, lectins and types II, III and VI secretion systems [11], [12], [19], [20], [21], [22], [23], [24], [25]. The *B. cenocepacia* CepIR system contributes to virulence in murine, *Caenorhabditis elegans*, *Galleria mellonella*, *Danio rerio* and alfalfa infection models [23], [26], [27], [28]. The CciIR system influenced virulence in the rat chronic respiratory infection model but did not affect virulence in *C. elegans* or *G. mellonella* [15], [27]. It has been shown that *B. cenocepacia* QS influences bacterial dissemination from initial infection sites in mouse and *D. rerio* infection models although the specific mechanism(s) have not been revealed [23], [28].

Despite having a detailed understanding of the core regulatory features that govern *B. cenocepacia* QS, to date only four additional regulators have been identified that induce or repress QS [29], [30]. The *B. cenocepacia* genome is of considerable size (>8 Mbp) and contains many uncharacterized genes. We previously showed CepR positively regulated expression of the BCAM1871 ORF located immediately downstream from *cepI* [19]. We hypothesized the conserved hypothetical protein encoded by BCAM1871was involved in *B. cenocepacia* virulence based on its genomic location, protein conservation and regulation by QS. In this study, we used gene expression and phenotypic assays as well as vertebrate and invertebrate infection models to assess the contribution of BCAM1871 to pathogenesis in *B. cenocepacia*.

## Results

### BCAM1871 is co-transcribed with *cepI* and directly regulated by CepR

**Figure 1:**
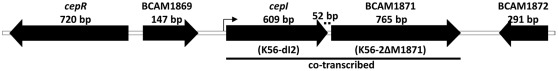
Genetic organization of the BCAM1871 locus. BCAM1871 is located downstream from *cepI* with 52 bp separating *cepI* and BCAM1871 which were determined to be co-transcribed (solid line) using RT-PCR. Size (bp) of each ORF is indicated. The *cepI* transcription start site is located 28 bp upstream of the ATG start codon [18]. One promoter (arrow) was identified for the *cepI*-BCAM1871 operon, and this was located upstream of *cepI* and included in the *cepI* promoter::*lux* fusion (pCP300). Nomenclature used to describe K56-dI2 and K56-2ΔBCAM1871 mutants.

In *B. cenocepacia* the *cepI* and *cepR* ORFs are divergently transcribed and separated by BCAM1869 (Fig. 1). Fifty-two bp downstream from *cepI* lies the BCAM1871 ORF that we previously showed was positively regulated by CepR using microarrays [19]. To further study the regulation of BCAM1871 we compared expression of BCAM1871 in the *cepI* mutant (K56-dI2) which contains a 290 bp deletion in *cepI* using qRT-PCR [12]. Expression of BCAM1871 was substantially reduced (31.8±10.4-fold) in the *cepI* mutant compared to that of wild type. When we exogenously added 300 ρM OHL to cultures of the *cepI* mutant BCAM1871 expression was almost fully restored to wild type levels in that is was reduced only 4.7±2.0-fold. We determined that *cepI* and BCAM1871 were co-transcribed using RT-PCR with a primer pair designed to span the intergenic region (Fig. 1). BCAM1871 expression appeared to be driven from the *cepI* promoter as no other promoter was identified upstream of the BCAM1871 ORF using promoter prediction software (www.softberry.com or http://www.fruitfly.org, data not shown). CepR directly binds the *cepI* promoter to positively regulate its transcription [18], [30]. Our data suggested CepIR-dependent regulation of BCAM1871occurred through CepR binding to the *cepI* promoter driving expression of the *cepI*-BCAM1871 operon.

Orthologs of the ORF encoding BCAM1871 are conserved in their genomic locations, in that each lies downstream from an AHL synthase, in at least six species of the Bcc (*B. ambifaria*, *B. dolosa*, *B. lata*, *B. multivorans*, *B. ubonensis and B. vietnamiensis*) as well as *Burkholderia pseudomallei* and *Burkholderia mallei* [31]. In an analysis of 1657 genomes, orthologs of BCAM1871 were found in 41 genomes, all of which belonged to *Burkholderia* spp. indicating this protein is unique to this genus [31]. BCAM1871 encodes a conserved hypothetical protein that has limited homology (E value of 4.55^−04^) to hydroxymethylglutaryl-CoA reductase [32]. BCAM1871 is predicted to encode a protein of 254 aa that has a cytoplasmic localization [31].

### BCAM1871 enhances AHL activity

**Figure 2:**
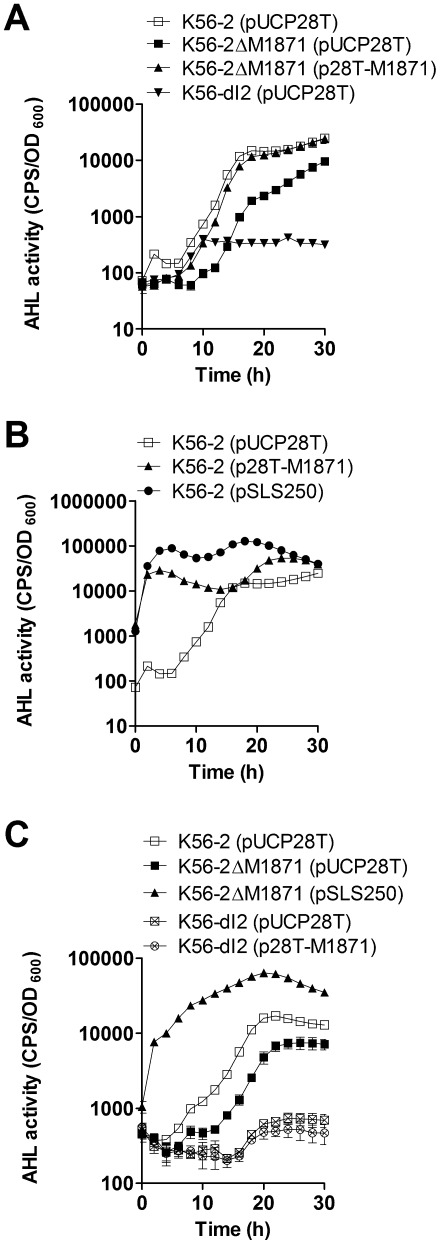
Influence of BCAM1871 on AHL activity. AHL activity was monitored using *A. tumefaciens* A136 (pCF218) (pMV26) in a real-time liquid co-culture assay. (A) Significantly decreased in K56-2ΔM1871 (pUCP28T) compared to that in K56-2 (pUCP28T) from 16–30 h. Significantly increased in K56-2ΔM1871 (p28T-M1871) compared to that in K56-2ΔM1871 (pUCP28T) from 18–30 h. Significantly decreased in K56-dI2 (pUCP28T) compared to that in K56-2ΔM1871 (pUCP28T) from 16–30 h. (B) Significantly increased in K56-2 (p28T-M1871) compared to that in K56-2 (pUCP28T) from 2–10 h and 18–30 h. Significantly increased in K56-2 (pSLS250) compared to that in K56-2 (p28T-M1871) from 4–25 h. (C) Significantly increased in K56-2ΔM1871 (pSLS250) compared to that in K56-2ΔM1871 (pUCP28T) from 2–30 h. No significant difference in K56-dI2 (pUCP28T) compared to that in K56-dI2 (p28T-M1871). All p values<0.001.

We hypothesized BCAM1871 may affect AHL activity based on its genomic location, protein conservation and regulation by QS. A BCAM1871 mutant was constructed by removing an internal 365 bp portion of the ORF. Mutation of BCAM1871 did not affect growth of the strain at 29°C or 37°C compared to wild type in LB (data not shown). Using a real-time co-culture AHL activity assay, the BCAM1871 mutant had significantly reduced AHL activity throughout growth compared to wild type (Fig. 2A). AHL activity was not restored to wild type levels when BCAM1871 was expressed on a lower copy plasmid (pM1871). This was likely due to insufficient expression of BCAM1871 which is expressed at relatively high levels in stationary phase in wild type (data not shown). Consistent with a relatively high expression level, AHL activity was restored to wild type levels by expression of BCAM1871 in *trans* on a high copy plasmid (p28T-M1871) (Fig. 2A). Mutation of BCAM1871 did not completely abolish AHL production. In fact, AHL activity of the BCAM1871 mutant was significantly higher than that observed for the *cepI* mutant (Fig. 2A).

Considering BCAM1871 mutation had a more subtle effect on AHL activity than a *cepI* mutation, we decided to examine potential effects of overexpressing BCAM1871 in wild type cells. Overexpression of BCAM1871 significantly enhanced AHL activity above wild type levels in early stages of growth, albeit to a lower level than observed for overexpression of CepI (Fig. 2B). These data suggested the effect of BCAM1871 on AHL activity was likely related to enhancing AHL production rather than being directly involved in AHL synthesis. Supporting the hypothesis that BCAM1871 indirectly affected AHL production, and with the predicted protein function indicating no relationship to AHL synthases, expression of BCAM1871 in *trans* in the heterologous host *E. coli* resulted in no detectable AHL activity. In contrast, high levels of AHL activity were observed when *cepI* was expressed in *E. coli* (data not shown). To further explore the AHL activity relationship between BCAM1871 and *cepI*, we performed cross-complementation experiments. AHL activity was increased in the BCAM1871 mutant expressing *cepI* in *trans* to levels higher than that those observed in wild type (Fig. 2C). In contrast, AHL activity was not altered in the *cepI* mutant expressing BCAM1871 in *trans* (Fig. 2C). We also sought to determine if a secreted factor(s) from wild type could fully restore AHL activity to the BCAM1871 mutant. AHL activity in a 1∶1 mixed culture of wild type and the BCAM1871 mutant was significantly increased compared to that in the BCAM1871 mutant but still significantly reduced compared to wild type (data not shown). These data suggested lower AHL activity in the BCAM1871 mutant was not attributable to absence of a secreted factor in this strain. Together, these data indicate BCAM1871 acts as an enhancer of AHL activity rather than being essential or sufficient for AHL activity in *B. cenocepacia*.

We considered the possibility that BCAM1871 could exert a regulatory influence in another bacterial species with an AHL-dependent QS system. There was no difference in AHL activity or expression of a *lasI*::*lux* fusion when *B. cenocepacia* BCAM1871 was expressed in *trans* in *Pseudomonas aeruginosa* PAO1 compared to vector control (data not shown). These data suggest the effect of BCAM1871 may not be observed in other organisms but may be specific to *Burkholderia* spp or *B. cenocepacia*.

### BCAM1871 promotes transcription of *cepIR* and *cciIR*

**Figure 3:**
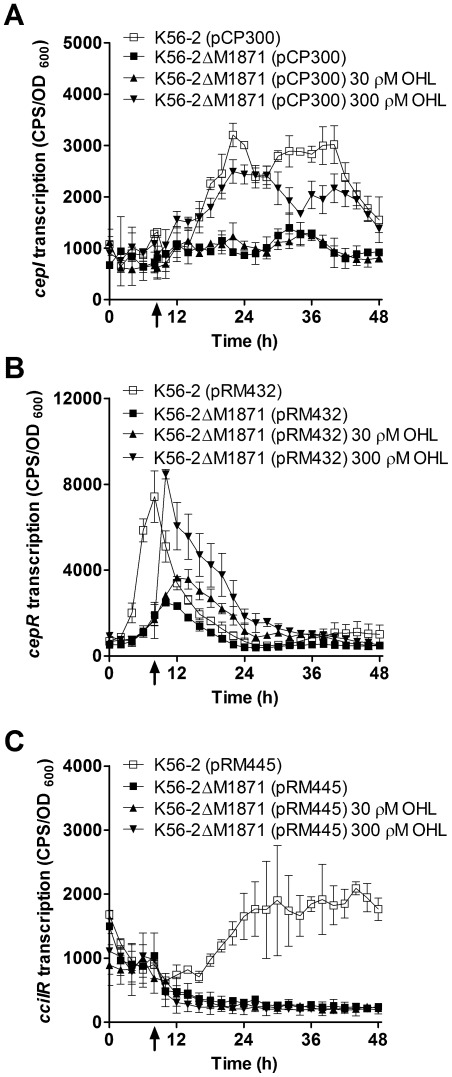
Transcription of *cepIR* and *cciIR*. Transcription was monitored using promoter::*lux* fusions in LB ± OHL at 37°C. OHL was added at 8 h (arrow). (A) *cepI* (pCP300) expression was: significantly decreased in K56-2ΔM1871 compared to that in K56-2 from 17.5–28.5 h (p<0.05); significantly increased in K56-2ΔM1871 300 ρM OHL compared to that in K56-2 from 19.5–30 h p<0.001 and 37.5–44 h (p<0.01). (B) *cepR* (pRM432) expression was: significantly decreased in K56-2ΔM1871 compared to that in K56-2 from 4–12.5 h (p<0.001); significantly increased in K56-2ΔM1871 30 ρM OHL compared to that in K56-2 from 11.5–22.5 h (p<0.01); significantly increased in K56-2ΔM1871 300 ρM OHL compared to that in K56-2 from 8.5–25 h (p<0.001). (C) *cciIR* (pRM445) expression was: significantly decreased in K56-2ΔM1871 compared to that in K56-2 from 22.5–48 h (p<0.001).

Since BCAM1871 appears to enhance AHL activity, the effects of BCAM1871 mutation on expression of promoter::*lux* fusions were determined to measure transcription of QS genes. Expression of *cepI*, *cepR* and *cciIR* was significantly reduced in the BCAM1871 mutant compared to wild type (Fig. 3). Addition of 300 ρM exogenous OHL to cultures immediately prior to maximal *cepR* promoter activity in wild type (8 h) resulted in significantly increased *cepI* and *cepR* expression in the BCAM1871 mutant (Fig. 3AB). In contrast, expression of *cciIR* was not restored to wild type levels at any point during growth of the BCAM1871 mutant in cultures supplemented with exogenous OHL (Fig. 3C). Addition of 30 ρM exogenous OHL had a significant effect on *cepR* but not *cepI* or *cciIR* transcription (Fig. 3). Furthermore, we found the timing of OHL addition was critical since supplementation with exogenous OHL at the start of the culture (0 h) had no effect on *cepI*, *cepR* or *cciIR* transcription (data not shown).

### BCAM1871 promoted motility independently of AHL activity or CepIR function

**Figure 4:**
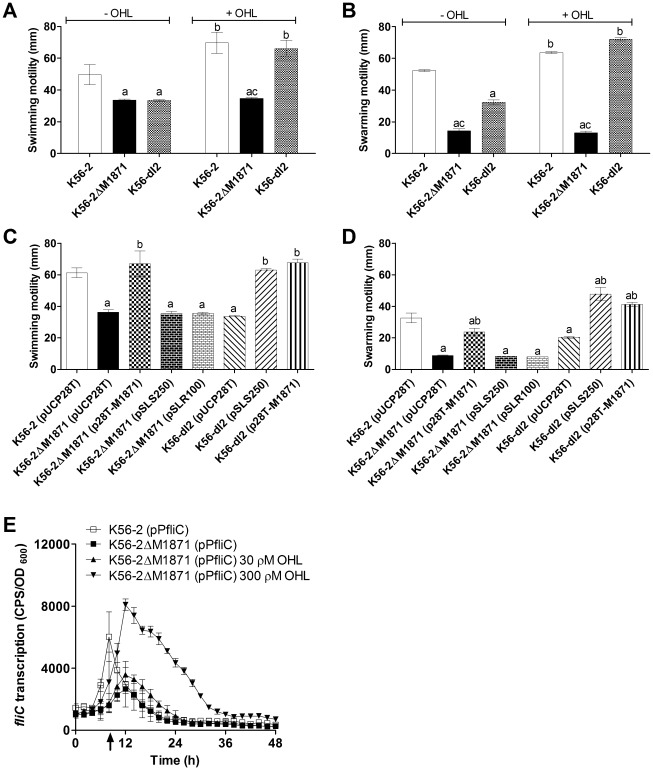
Motility and transcription of *fliC*. Swimming and swarming motility assays were performed using L-agar plates supplemented with 0.25% and 0.5% agar, respectively, ±300 ρM OHL. (A) and (C) Swimming motility at 24 h. (B) and (D) Swarming motility at 40 h. (A) and (B) Significantly different compared to: a, K56-2; b, without OHL; c, K56-dI2. (C) and (D) Significantly different compared to: a, K56-2 (pUCP28T); b, appropriate parent strain K56-2ΔM1871 (pUCP28T) or K56-dI2 (pUCP28T). All p values <0.001. (E) Transcription was monitored using promoter::*lux* fusions in LB ± OHL at 37°C. OHL was added at 8 h (arrow). *fliC* expression was: significantly decreased in K56-2ΔM1871 compared to that in K56-2 from 6–10 h (p<0.01); significantly increased in K56-2ΔM1871 30ρM OHL compared to that in K56-2 from 8.5–18.5 h (p<0.01); significantly increased in K56-2ΔM1871 300ρM OHL compared to that in K56-2 from 8.5–28.5 h (p<0.001).

A number of phenotypic traits are controlled by QS in *B. cenocepacia* including bacterial motility and production of extracellular enzymes. We previously showed motility is reciprocally regulated by CepIR and CciIR in *B. cenocepacia* [19]. Different modes of motility, such as swimming and swarming, can be differentiated using L-agar plates supplemented with 0.25% and 0.5% agar, respectively. In each mode of motility, the BCAM1871 mutant showed significantly reduced translocation compared to that of wild type (Fig. 4AB). Furthermore, the BCAM1871 mutant was unresponsive to exogenous OHL in terms of motility. A significant reduction in swimming and swarming motility was observed for the *cepI* mutant compared to wild type (Fig. 4AB), adding to and confirming previous data that showed reduced swarming motility [12] for an independently constructed *cepI* insertion mutant (K56-I2) [11]. Addition of exogenous OHL to the medium significantly stimulated motility in wild type and restored motility to wild type levels in the *cepI* mutant, in contrast to the unresponsive BCAM1871 mutant (Fig. 4AB). We also noted differences between the *cepI* and BCAM1871 mutants in that swarming motility, but not swimming motility, was significantly decreased in the BCAM1871 compared to the *cepI* mutant in absence of OHL (Fig. 4AB).

Reduced swimming and swarming motilities of the BCAM1871 mutant were significantly increased by expressing BCAM1871 in *trans*, confirming the involvement of BCAM1871 in regulating this motility phenotype (Fig. 4CD). In a series of cross-complementation experiments, we found expressing BCAM1871 in *trans* in the *cepI* mutant significantly increased motility to levels similar to that seen for expression of *cepI* in *trans* in the *cepI* mutant (Fig. 4CD). We also employed the alternative strategy of introducing a *cepI* expression construct into the BCAM1871 mutant. Expression of *cepI* in *trans* did not alter the motility defect of the BCAM1871 mutant corroborating data above that showed the BCAM1871 mutant was unresponsive to exogenous OHL in terms of motility (Fig. 4CD). Furthermore, expression of *cepR* in *trans* in the BCAM1871 mutant had no effect on motility in medium with or without exogenous OHL (Fig. 4CD and data not shown).

Swimming and swarming motility is influenced by flagellar function. Expression of *fliC*, encoding flagellin, was significantly reduced (up to 15-fold) in the BCAM1871 mutant compared to wild type (Fig. 4E). Addition of 30 or 300 ρM exogenous OHL significantly increased *fliC* expression in the BCAM1871 mutant to wild type levels or greater (Fig. 4E) despite having no effect on the reduced motility phenotype described above (Fig. 4CD). Together these data show that the BCAM1871 mutant was significantly affected for motility and that exogenous OHL or CepIR function was insufficient to alter the reduced motility phenotype of the BCAM1871 mutant.

### BCAM1871 promoted protease activity independently of AHL activity or CepIR function

**Figure 5:**
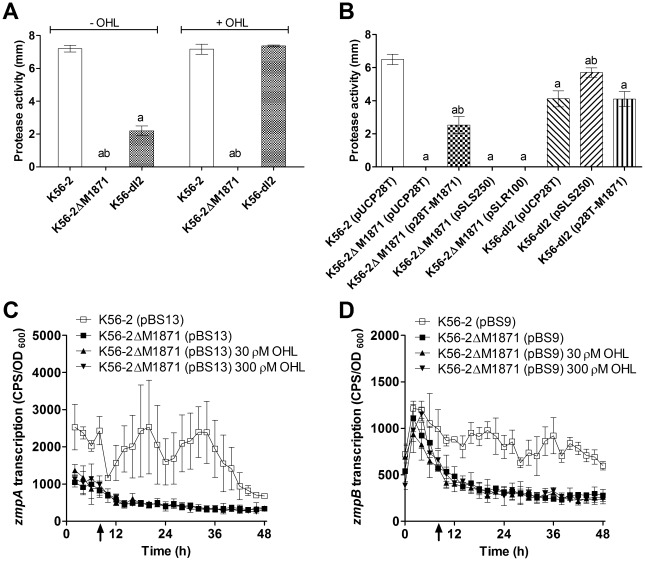
Protease activity and transcription of *zmpA* and *zmpB*. Protease activity was determined using D-BHI with 1.5% skim milk agar plates after 40 h incubation at 37°C, ±2500 ρM OHL. (A) Significantly different compared to: a, K56-2; b, K56-dI2. (B) Significantly different compared to: a, K56-2 (pUCP28T); b, appropriate parent strain K56-2ΔM1871 (pUCP28T) or K56-dI2 (pUCP28T). All p values <0.001. Transcription was monitored using promoter::*lux* fusions in LB ± OHL at 37°C. OHL was added at 8 h (arrow). (C) *zmpA* (pBS13) expression was: significantly decreased in K56-2ΔM1871 compared to that in K56-2 from 14–14.5, 16–22 and 27.5–37.5 h (p<0.05). (D) *zmpB* (pBS9) expression was: significantly decreased in K56-2ΔM1871 compared to that in K56-2 from 11.5–48 h (p<0.05).

*B. cenocepacia* possesses a T2SS that is responsible for the secretion of several extracellular enzymes such as zinc metalloproteases ZmpA and ZmpB that influence virulence in a rat chronic respiratory infection model [22], [33]. Expression of *zmpA* and *zmpB* is carefully regulated by CepIR and CciIR leading us to hypothesize that AHL activity differences in the BCAM1871 mutant may also affect protease activity. We were unable to detect protease activity surrounding cultures of the BCAM1871 mutant on skim milk agar up to 40 h incubation and this phenotype was not altered by exogenous OHL (Fig. 5A). Additionally, we observed the BCAM1871 mutant had a more severe protease activity defect than previously observed for the *cepI* mutant, from which some protease activity was detected (Fig. 5A), albeit at significantly reduced levels compared to wild type as previously determined [11]. Supplementation of the medium with OHL restored protease activity to wild type levels in the *cepI* mutant as expected (Fig. 5A).

Expression of BCAM1871 in *trans* significantly enhanced protease activity in the BCAM1871 mutant but did not affect protease activity in the *cepI* mutant (Fig. 5B). Confirming these data, expression of BCAM1871 in *trans* did not affect protease activity in an independently constructed *cepI* insertion mutant (K56-I2) (data not shown). Significantly increased protease activity was detected by expressing *cepI* in *trans* in the *cepI* mutant. However, expressing *cepI* in *trans* in the BCAM1871 mutant had no effect on its protease negative phenotype (Fig. 5B) confirming the results above obtained with addition of exogenous OHL to the medium (Fig. 5A). Expression of *cepR* in *trans* in the absence or presence of OHL also did not affect the protease negative phenotype of the BCAM1871 mutant (Fig. 5B and data not shown). Together, these data suggest BCAM1871 and CepIR independently promote protease activity.

Consistent with the protease negative phenotype of the BCAM1871 mutant, expression of *zmpA* and *zmpB* was reduced to background levels (i.e. similar to the promoterless *lux* fusion plasmid pMS402) in the BCAM1871 mutant compared to that of wild type (Fig. 5CD). Consistent with the phenotypic data, expression of *zmpA* and *zmpB* was unaffected by addition of exogenous OHL to the medium (Fig. 5CD). Since addition of exogenous OHL to the medium, or expression of *cepI* or *cepR* in *trans* in the BCAM1871 mutant, did not alter either motility or protease activity, it suggests that unidentified factor(s) controlled by BCAM1871, other than AHL activity or CepIR function, are required for expression of these phenotypes in *B. cenocepacia*.

### BCAM1871 influences expression of additional virulence-related genes

**Figure 6:**
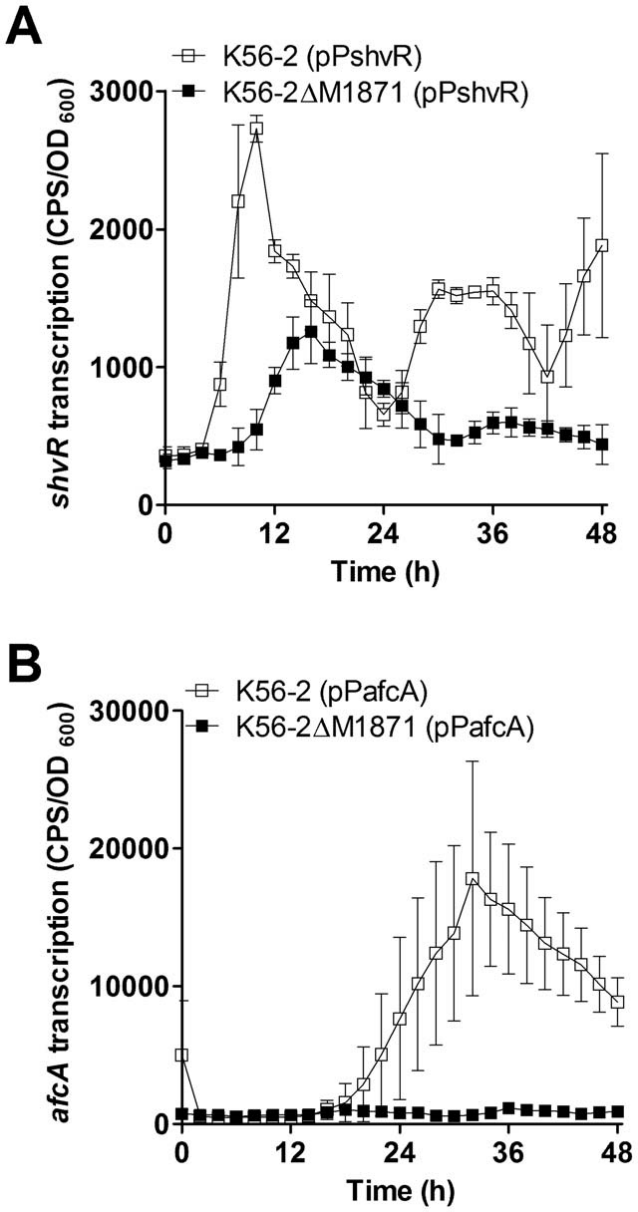
Transcription of *shvR* and *afcA*. Transcription was monitored using promoter::*lux* fusions in LB at 37°C. (A) *shvR* expression was significantly decreased in K56-2ΔM1871 compared to that in K56-2 from 7–13 h and 28.5–38.5 h (p<0.001). (B) *afcA* expression was significantly decreased in K56-2ΔM1871 compared to that in K56-2 from 26–44 h (p<0.001).

Our laboratory is also interested in other *B. cenocepacia* genes that contribute to virulence. The *B. cenocepacia* LysR-type transcriptional regulator, ShvR, influences colony morphology, biofilm formation, cell surface hydrophobicity, QS, type II secretion, antifungal activity, as well as plant and animal virulence [30], [34], [35]. Some of these characteristics are controlled through direct regulation by ShvR of the adjacent *afcA* operon that contains twenty-two genes, some of which influence colony morphology, biofilm formation and virulence [35]. Expression of *shvR* and the *afcA* operon was significantly decreased in the BCAM1871 mutant compared to wild type (Fig. 6), in a manner similar to that observed for indirect CepR regulation of *shvR* and *afcA* [30].

### BCAM1871 contributes to persistence and inflammation in a rat chronic respiratory infection model

**Figure 7:**
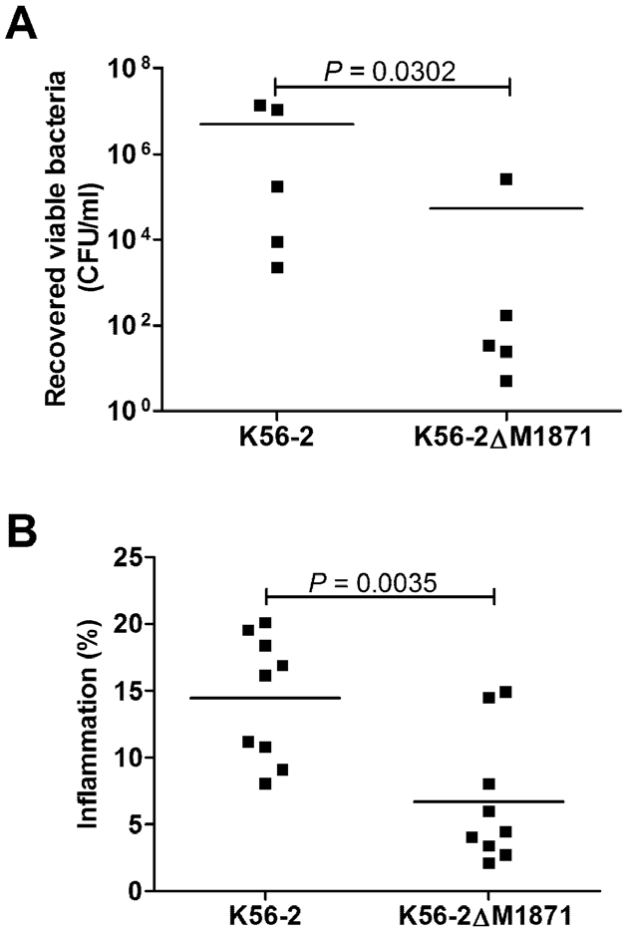
Bacterial persistence and inflammation in a rat chronic respiratory infection model. At seven days postinfection rat lungs were harvested and used for (A) quantitative bacteriology by plating lung homogenates and determining the number of colony forming units or (B) quantitative histopathology analysis of hematoxylin and eosin stained lung sections. Results are displayed using scatter plots with mean values represented by the horizontal bars. P values indicate significant differences in lungs infected with K56-2ΔM1871 compared to that in K56-2.

Considering the effect of mutating BCAM1871 on the above virulence-related phenotypes and gene expression we chose to assess the contribution of BCAM1871 to virulence in a rat chronic respiratory infection model. Rats were infected with approximately 10^8^ CFU of the wild type and BCAM1871 mutant. The number of bacteria recovered from lungs seven days post infection was significantly reduced in animals infected with the BCAM1871 mutant compared to wild type indicating the BCAM1871 mutant had reduced ability to persist in the lungs (Fig. 7A). Lung sections were obtained and stained with hematoxylin and eosin to quantify pathological changes associated with infection. Consistent with reduced bacterial burden, significantly reduced inflammation was observed in rats infected with the BCAM1871 mutant compared to wild type (Fig. 7B).

### The BCAM1871 mutant displayed reduced expression of a gene encoding a nematocidal protein and caused reduced nematode death

**Figure 8:**
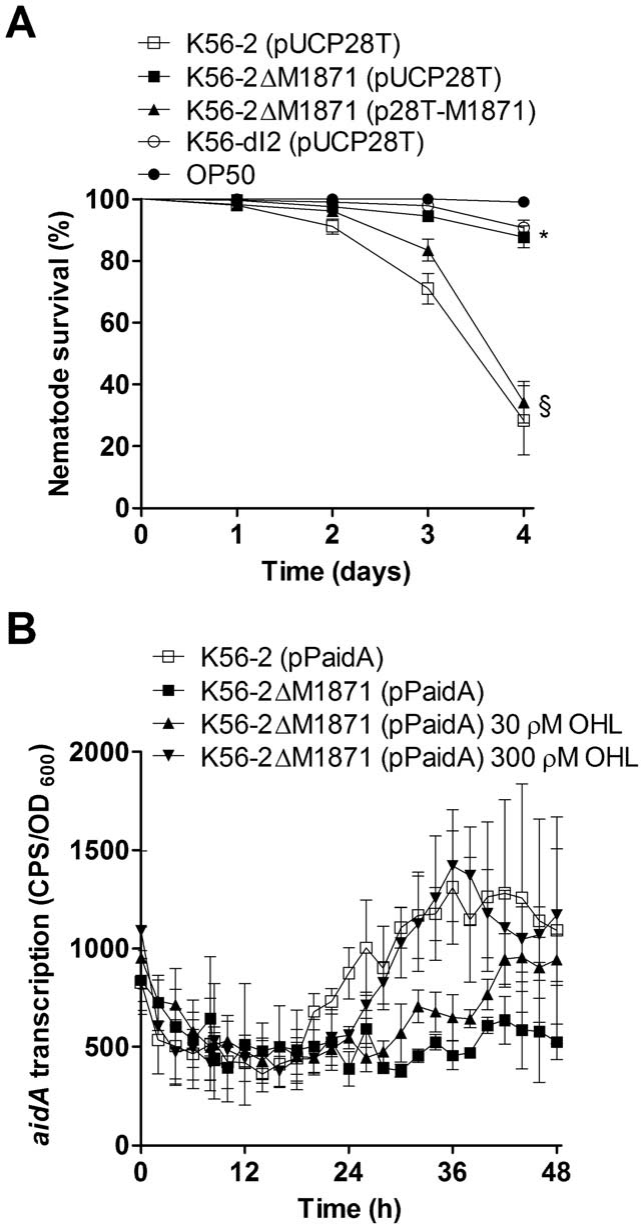
Nematode survival and transcription of *aidA*. (A) Nematode survival was monitored by analyzing response to touch after feeding on strains indicated. *, significantly decreased in K56-2ΔM1871 (pUCP28T) or K56-dI2 (pUCP28T) compared to that in K56-2 (pUCP28T) (p<0.0001). §,significantly increased in K56-2ΔM1871 (p28T-M1871) compared to that in K56-2ΔM1871 (pUCP28T) (p<0.0001). Values are the means ± standard error of results from plates containing at least 25 worms per plate and are representative of results from at least two individual trials. (B) Transcription was monitored using promoter::*lux* fusions in LB ± OHL at 37°C. OHL was added at 8 h (arrow). *aidA* expression was: significantly decreased in K56-2ΔM1871 compared to that in K56-2 from 29.5–42.5 h (p<0.05) (most timepoints); significantly increased in K56-2ΔM1871 300 ρM OHL compared to that in K56-2 from 30–41 h (p<0.001).

AidA is a relatively uncharacterized protein in *B. cenocepacia* that significantly contributes to virulence of the nematode *C. elegans* [36]. Nematode survival was significantly higher following feeding infection with the BCAM1871 or *cepI* mutant compared to feeding infection with wild type (Fig. 8A). Expression of BCAM1871 in *trans* significantly decreased nematode survival to a level similar to that observed for wild type (Fig. 8A). Along with *cepI*, CepR-dependent expression of *aidA* has been consistently identified in numerous studies [18], [19], [37], [38]. We found *aidA* transcription was significantly decreased in the BCAM1871 mutant compared to wild type and its expression was significantly influenced by 300 ρM exogenous OHL in the medium (Fig. 8B).

## Discussion

**Figure 9:**
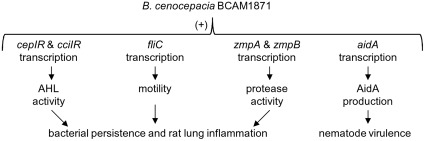
Effects of BCAM1871 on transcription, phenotype and virulence of *B. cenocepacia*. BCAM1871 positively (+) influences transcription of several genes invoved in altering phenotypes that contribute to pathogenesis in rat and nematode infection models.

In this study, we have characterized multiple deleterious effects of mutating the ORF encoding BCAM1871 on pathogenesis-related traits in *B. cenocepacia*. Our data suggests BCAM1871 is an integral component of the *B. cenocepacia* QS network and its inactivation disrupts optimal timing and magnitude of cell-density dependent gene expression and virulence factor production, resulting in significantly reduced pathogenesis in rat and nematode infection models (Fig. 9).

The predicted protein encoded by BCAM1871 appears to be unique and conserved in its genetic context with *cepIR* homologs in *Burkholderia* spp suggesting it has been conserved evolutionarily and retains an important function. In the predicted protein sequence for BCAM1871, there exists a partial domain with similarity to hydroxymethylglutaryl-CoA reductases (HMGR) (e value 4.6^−4^) despite absence of an N-terminal membrane region. HMGR function as rate-limiting enzymes in cholesterol biosynthesis in eukaryotes (class I) and isoprenoid biosynthesis in prokaryotes (class II) [39]. *B. cenocepacia* contains a putative class II HMGR encoded by *mvaA* (BCAM0531) that has little or no similarity at the amino acid level to BCAM1871 (data not shown). Our attempts to inhibit activity of these HMGR using mevastatin [39] had no effect on AHL or protease activity of *B. cenocepacia* (data not shown).

Apart from the synthases and transcriptional regulators that form the core of the *B. cenocepacia* AHL-mediated QS system, only four regulators were previously demonstrated to positively or negatively influence *B. cenocepacia* QS. Three regulators were identified that promoted AHL activity and biofilm formation in *B. cenocepacia* H111 [29]. These mutants had defects in biofilm formation that were not restored by exogenous OHL. Furthermore, the mutants exhibited defects in biofilm formation and AHL activity that could not be rescued by expressing *cepR* in *trans* [29]. Based on similarities in results obtained for these regulators and BCAM1871, it appears each of them exerts activity upstream of CepR in the *B. cenocepacia* QS hierarchy, although we additionally demonstrated *cepR* is affected at the transcriptional level by BCAM1871 mutation. We also determined mutation of BCAM1871 had no effect on biofilm formation (data not shown) indicating additional differences upon mutation of the three previously described regulators [29] or BCAM1871. Our results showed BCAM1871 is positively autoregulated by enhancing AHL activity and *cepIR* expression which in turn influences CepR-dependent regulation of the *cepI*-BCAM1871 operon. In addition to these positive regulators, we showed mutation of BCAM1871 reduced expression of the LysR-type transcriptional regulator ShvR. ShvR is a negative regulator of *cepIR* and *cciIR* transcription that affects AHL activity and secretion of virulence factors [30]. Our laboratory previously showed ShvR was important for biofilm formation, colony morphology and virulence in plant and animal infection models [34]. The fact that ShvR and CepR independently control biofilm formation [30], and that mutation of BCAM1871 had no effect on biofilm formation, despite reduced *shvR* and *cepR* expression, suggests additional factors regulating biofilm formation in *B. cenocepacia* have yet to be uncovered.

We observed a more severe defect in the BCAM1871mutant compared to the *cepI* mutant for swarming motility and protease activity despite the fact that BCAM1871 mutant retains more AHL activity than the *cepI* mutant. Previous studies attributed defects in motility, protease activity or *zmpA/zmpB* gene expression to mutation of *cepR* or *cepI* which could be complemented by providing *cepR* or *cepI* in *trans*, or by supplying exogenous OHL [11], [12], [21], [23], [24]. The current study refines our understanding to show that *cepR* or *cepI* mutation would in fact have adversely affected expression of the *cepI*-BCAM1871 operon. The transcriptional coupling of *cepI* and BCAM1871 means that BCAM1871 expression is reduced, but not abolished, in a *cepI* mutant and can be rescued to near wild type levels by expressing *cepI* in *trans* or by providing exogenous OHL. We also observed that significantly reduced transcription of BCAM1871 in a *cepIcciI* mutant was increased by providing exogenous OHL, but not HHL (data not shown). In contrast, reduced motility or protease activity in the BCAM1871 mutant could not be altered by providing *cepI* or *cepR* in *trans*, or by supplying exogenous OHL, but only by expressing BCAM1871 in *trans*.

We cannot fully explain why exogenous OHL stimulated *fliC* transcription but neither swimming nor swarming motility. This result could be due to deregulation of one or more of the approximately thirty flagellar-associated motility genes or the production of a biosurfactant that has not yet been identified in *B. cenocepacia*. Exogenously-supplied biosurfactants restored swarming motility to a *B. cenocepacia* H111 *cepR* mutant [21]. The positive effect of OHL-mediated QS on indirect regulation of flagellar-associated motility has been studied in more detail in the rice plant pathogen *Burkholderia glumae* [40]. In fact, lack of flagella formation in a *B. glumae* AHL synthase (*tofI*) mutant can be overcome by incubation at 28°C indicating other factors besides QS are involved [40]. We previously found reciprocal regulation of swimming motility by CepR and CciR was apparent at 22°C and 28°C [19]. It was interesting to note that expressing BCAM1871 in *trans* promoted motility but did not affect protease activity in *cepI* mutant. These different outcomes on motility and protease activity in the *cepI* mutant occurred despite the fact that expressing BCAM1871 in *trans* does not affect AHL activity of the *cepI* mutant, indicating BCAM1871 can affect motility independently of altering AHL activity in this strain. We also demonstrated addition of exogenous OHL to cultures of the wild type promoted motility but not protease activity. This suggests protease activity is controlled by factors in addition to QS or the effects of QS can be saturated or subject to negative feedback by CciIR as previously described [19], [22]. Together, these data suggest there are additional factors directly or indirectly affected by BCAM1871 mutation that regulate these genes and the phenotypes they affect.

Upon exogenous OHL supplementation, different responses were observed in the BCAM1871 mutant with respect to transcription of affected genes. For the most part, exogenous supplementation with 300 ρM, but not 30 ρM, OHL was sufficient to restore expression of those genes that were responsive to exogenous OHL in the BCAM1871 mutant. It was previously shown that *cepR* expression was increased in a *cepR* mutant using a promoter::*lacZ* fusion indicating *cepR* was negatively autoregulated [11]. In our study, *cepR* transcription could be increased by exogenous OHL supplementation immediately prior to maximal *cepR* transcription using a *cepR::lux* fusion in wild type (data not shown) or the BCAM1871 mutant. It appears that optimal timing of OHL supplementation is critical since these effects were not observed for wild type or the BCAM1871 mutant when exogenous OHL was added at the start of the culture (data not shown). Consistent with increased *cepR* transcription upon exogenous OHL supplementation in the BCAM1871 mutant, we also observed increased expression of two genes (*cepI* and *aidA*) that are directly regulated at the promoter level by CepR [18]. Although it has not been demonstrated that CepR-dependent regulation occurs at the promoter level for *fliC*, we observed *fliC* transcription was restored by exogenous OHL in the BCAM1871 mutant. In contrast, exogenous OHL had no effect on transcription of *zmpA*, *zmpB* and *cciIR*. It is noteworthy that *zmpA* is not directly regulated at the promoter level by CepR [41], and *zmpB* and *cciIR* do not contain a *cep* box motif in their promoters [20] suggesting they are also not directly regulated by CepR. We did not investigate potential effects on posttranscriptional control of *cepR*, stability of CepR or its ability to bind and retain OHL since mutation of BCAM1871 had a significant effect on *cepR* transcription.

The BCAM1871 mutant had a similar effect on nematode survival and *aidA* gene expression as previously demonstrated for a *cepI* mutant [26], [36]. In the rat chronic respiratory infection model, reduced persistence was observed for the BCAM1871 mutant at seven days postinfection which was not identified for *cepI* or *cepR* mutants at ten days postinfection [23]. Despite decreased lung persistence we detected no defect in intracellular survival of the BCAM1871 mutant in RAW264.7 murine macrophages or in resistance to buffered solutions at low pH (6.4 or 4.5) (data not shown), i.e. pH values that have been quantified inside macrophages [42].

Recent studies identified a small regulatory protein CinS that is cotranscribed with the AHL synthase CinI in *R. leguminosarum* [43]. In contrast to the effect of BCAM1871 on *cepI* transcription, mutation of *cinS* did not affect *cinI* transcription or synthesis of the CinI gene product, but resulted in reduced expression of a second AHL synthase, RaiI, and the RaiI gene product [43]. A swarming defect in *R. leguminosarum* that was attributed to *cinI* mutation [44] was in fact due to polar effects of *cinI* mutation on *cinS* expression [43]. These swarming defects in *R. leguminosarum cinI* or *B. cenocepacia cepI* mutants can each be restored by expressing the downstream gene (*cinS* or BCAM1871, respectively) in *trans*. This indicates the primary defect in these strains results from lower expression of the downstream gene, through effects of the *cinI*-*cinS* or *cepI*-BCAM1871 operons. Further work determined CinS interacted with an AHL-independent transcriptional repressor PraR to relieve the effects of this repressor on the *raiIR* and *rhiIR* QS systems in *R. leguminosarum* [45]. The predicted protein product of BCAM1871 (254 residues) does not show homology to CinS (67 residues), and neither CinS nor RpaR homologs are found in *B. cenocepacia* (data not shown).

Further experiments are required to characterize the mechanism of action of the regulator encoded by BCAM1871 which could include DNA-, RNA- or protein-binding capabilities. Such regulatory mechanisms have previously been identified for other QS systems that lead to modulation of QS activity thereby enabling fine-tuned control of gene expression (for review see, [46]–[49]). Mutation of BCAM1871 resulted in significant effects on gene expression, phenotypes and virulence. While BCAM1871 is an enhancer of AHL synthesis and it transcription is CepIR-regulated, some phenotypes were affected independently of AHL activity or CepIR function suggesting BCAM1871 operates by a novel mechanism that has not yet been described elsewhere and potentially involves a sophisticated coordination of AHL-dependent and AHL-independent activities.

## Materials and Methods

### Strains, plasmids and growth conditions

The bacterial strains and plasmids used in this study are listed in Table 1. Cultures were routinely grown at 37°C, in Miller's Luria broth (LB) (Invitrogen, Burlington, ON) with shaking or on 1.5% Lennox LB agar plates. For RT-PCR or qRT-PCR experiments, cultures were grown to stationary phase (16 h) in 10 ml LB in 125 ml erlenmeyer flasks. For promoter::*lux* assays, strains were grown in LB or trypticase soy broth (TSB) (Difco, Franklin Lakes, NJ) in black, clear-bottom, 96-well plates (Corning, Inc., Corning, NY) and assays performed as previously described [19], [20]. Cultures entered stationary phase at 18 h (growing at 37°C) or 30 h (growing at 29°C) [30]. For selected qRT-PCR or promoter::*lux* experiments, OHL (dissolved in 20% acetonitrile), was added in concentrations ranging from 30–300 ρM and results compared to addition of solvent control. AHL activity was monitored using *Agrobacterium tumefaciens* A136 (pCF218) (pMV26) in a real-time liquid co-culture assay with *B. cenocepacia* or *P. aeruginosa* grown in 96-well plates in TSB at 29°C [50]. Swimming and swarming motility assays were performed using L-agar plates supplemented with 0.25% and 0.5% agar, respectively [51]. Overnight cultures were normalized to an OD_600_ of 0.4 prior to inoculation and growth was assessed at 37°C. Protease activity was determined using D-BHI with 1.5% skim milk agar plates [52] using strains grown in 0.25% trypticase soy broth (Difco) with 5% Bacto-Peptone (Difco) (PTSB). Cell pellets were washed twice with PTSB and normalized to an OD of 0.3 prior to inoculation [30]. When appropriate, 2500 ρM exogenous OHL was spread on the surface of the protease agar and briefly air-dried prior to inoculation. When appropriate, the following concentrations of antibiotics were used: 100 µg/ml of trimethoprim (Tp) or 200 µg/ml tetracycline (Tc) for *B. cenocepacia*; 200 µg/ml Tc for *P. aeruginosa*; 100 µg/ml of Tp and 50 µg/ml of kanamycin (Km) for *E. coli*; and 25 µg/ml of Km and 4.5 µg/ml of Tc for *A. tumefaciens.* Antibiotics were purchased from Sigma-Aldrich Canada Ltd. (Oakville, ON).

**Table 1:** Bacterial strains and plasmids used in this study.

Strain or plasmid	Description	Reference
Strains		
*A. tumefaciens*		
A136	Ti plasmidless host	C. Fuqua
*E. coli*		
TOP10	F^−^ *mcrA*Δ (*mrr-hsd*RMS-*mcr*BC) φ80lacZΔM15 ΔlacX74 *deo*R *rec*A1 *ara*D139 Δ(ara-leu)7697 *gal*U *gal*K *rps*L *(St^R^)* endA1 *nupG*	Invitrogen
DH10B	F^−^ *mcr*A Δ(*mrr-hsd*RMS-*mcr*BC) φ80*lac*ZΔM15 Δ*lac*X74 *rec*A1 *end*A1 *ara*D139 Δ(*ara*, *leu*)7697 *gal*U *gal*K λ- *rps*L *nup*G	Invitrogen
SY327	*araD*, Δ*(lac pro) argE(Am) recA56 rif^R^nalA*λ*pir*	[62]
*B. cenocepacia*		
K56-2	CF isolate	[63]
K56-dI2	Δ*cepI* derivative of K56-2, internal 290 bp fragment removed	[12]
K56-I2	*cepI*::Tp derivative of K56-2, Tp^R^, (Tp cassette insertion)	[11]
K56-2ΔM1871	ΔM1871 derivative of K56-2, internal 365 bp fragment removed	This study
*P. aeruginosa*		
PAO1	Wild type	[64]
Plasmids		
pCR®2.1Topo	Cloning vector for PCR products, Ap^R^, Km^R^	Invitrogen
pCF218	IncP plasmid expressing TraR, Tc^R^	[65]
pMV26	*traI-luxCDABE* fusion; Km^R^	[23]
pRK2013	ColEl Tra (RK2)^+^, Km^R^	[66]
pGP*I-Sce*I	*ori*_R6K_, Tp^R^, *mob* ^+^, carries *I-Sce*I endonuclease recognition site, Tp^R^	[57]
pDA*I-Sce*I	pDA17 carrying the *I-Sce*I gene, Tc^R^	[57]
pGP*I-Sce*I-ΔM1871	pGP*I-Sce*I carrying BCAM1871 with a 365 bp internal fragment removed, Tp^R^	This study
pUCP28T	Broad-host-range vector, Tp^R^	[67]
pBBR1MCS-3	Broad-host-range vector, Tc^R^	[68]
pM1871	pBBR1MCS-3 with 0.9-kb *Kpn*I-*Apa*I fragment containing BCAM1871 coding region; Tc^R^	This study
p28T-M1871	pUCP28T with 0.9-kb *Kpn*I-*Apa*I fragment containing BCAM1871 coding region; Tp^R^	This study
pSLS250	pUCP28T with 1.5-kb *Sph*I-*Kpn*I fragment containing *cepI*; Tp^R^	[69]
pSLR100	pUCP28T with 1.65-kb *Kpn*I-*Sph*I fragment from pSLA3.2 containing *cepR*, Tp^R^	[11]
pMS402	*lux*-based promoter reporter plasmid, Km^R^ Tp^R^	[70]
pCP300	*cepI*::*lux* transcriptional fusion constructed in pMS402, Km^R^,Tp^R^	[12]
pRM432	*cepR*::*lux* transcriptional fusion constructed in pMS402, Km^R^,Tp^R^	[12]
pRM445	*cciIR*::*lux* transcriptional fusion constructed in pMS402, Km^R^,Tp^R^	[12]
pP*fliC*	*fliC*::*lux* transcriptional fusion constructed in pMS402, Km^R^, Tp^R^	This study
pP*aidA*	*aidA*::*lux* transcriptional fusion constructed in pMS402, Km^R^,Tp^R^	[20]
pBS13	*zmpA*::*lux* transcriptional fusion constructed in pMS402, Km^R^,Tp^R^, Tc^R^	[13]
pBS9	*zmpB*::*lux* transcriptional fusion constructed in pMS402, Km^R^,Tp^R^, Tc^R^	[22]
pP*shvR*	*shvR*::*lux* transcriptional fusion constructed in pMS402, Km^R^, Tp^R^	[30]
pP*afcA*	*afcA*::*lux* transcriptional fusion constructed in pMS402, Km^R^,Tp^R^	[19]
pP*lasI*	*lasI*::*lux* transcriptional fusion constructed in pMS402 and chromosomally integrated using mini-CTX*lux*, Km^R^,Tc^R^	[71]

### DNA manipulations

DNA manipulations were performed using standard techniques as described [53] and genomic DNA was isolated as described [54]. Oligonucleotide primers (Table 2) were designed with Primer3 [55] and were synthesized by the University of Calgary Core DNA and Protein Services (Calgary, Alberta, Canada). Plasmids were introduced into *B. cenocepacia* by electroporation [56].

**Table 2:** Oligonucleotide primers used in this study.

Primer	Sequence (5′-3′)^a^	Use	Reference
F1ΔM1871D1	GCTTGTCTTTCGCGCGATGATG	5′ BCAM1871 fragment for mutant construction	This study
R1ΔM1871*Cla*ID1	GCCTATCGATAGCACAGCGGCTCGTC		
F2ΔM1871*Cla*ID2	GCATATCGATATGACGAGGTCGGCGTG	3′ BCAM1871 fragment for mutant construction	This study
R2ΔM1871D2	GCAAGGCAAACGCGCCAAA		
M1871Dfor1	GGGGTACCCCTGAGCCGGAACGCGCG	Amplify entire BCAM1871 ORF	This study
M1871Drev2	GGGGGCCCTCTGCACAACTTGCCGCCGC		
*cepI*Dfor1	AGGTAGATGGGCGTCTGGT	Cotranscription of *cepI*-BCAM1871	This study
M1871Drev1	CTGCATCGTCAGGTCGTG		
M1871qRTfor1	CGGACTGGCTCGAATATCAC	BCAM1871 expression (qRT-PCR)	This study
M1871qRTrev1	GACCTCGTCATCGTCAACCT		
sigEqRTfor1	AGGAAACCAACCGTCAGATG	Housekeeper	[13]
sigEqRTrev1	GCGACGGTATTCGAACTTGT	(qRT-PCR)	

a Restriction enzyme sites underlined.

### Construction of the K56-2ΔM1871 mutant

The K56-2ΔM1871 mutant was constructed following a described method [57] using primers listed in Table 2. PCR confirmed deletion of a 365 bp fragment from the BCAM1871 ORF in this mutant.

### RNA manipulations and Quantitative RT-PCR (qRT-PCR)

RNA samples were prepared using a RiboPure bacterial RNA isolation kit (Ambion, Streetsville, Ontario, Canada) from *B. cenocepacia* strains grown to stationary phase in LB [19]. DNase treatment was performed, and samples were confirmed to be free of DNA by PCR using *Taq* polymerase (Invitrogen) prior to cDNA synthesis using an iScript Select cDNA synthesis kit (Bio-Rad, Mississauga, ON). Quantification and melting curve analyses were performed with iQ SYBR green Supermix on an iCycler (Bio-Rad) according to manufacturer's instructions. qRT-PCRs were performed in triplicate and normalized to the control gene, *sigE* (BCAM0918) as described previously [13]. Data were calculated as previously described [58] and data shown below are from three independent experiments.

### Animal studies

Animal infections were performed using the rat agar bead respiratory infection model [59]. Adult male Sprague-Dawley rats (150–180 g; Charles River, QC, Canada) were inoculated transtracheally with approximately 10^8^ CFU of the appropriate strain. At seven days postinfection, infected lungs were aseptically removed, and lungs from four to five animals per group were used for quantitative bacteriology or histopathology analysis as previously described [51]. Infiltration of hematoxylin-and-eosin-stained lung sections with inflammatory cells and exudates was quantitated using Image Pro Plus (Media Cybernetics, Bethesda, MD). Animal experiments were conducted according to the guidelines of the Canadian Council of Animal Care for the care and use of experimental animals under protocol M08089 approved by the University of Calgary Animal Care Committee.

### Nematode studies

Nematode infections were performed using *C. elegans* strain Bristol N2 as previously described [26]. Briefly, nematodes were maintained on nematode growth (NG) agar [60] with *Escherichia coli* OP50 as a food source at 25°C in a humidified atmosphere [61]. Killing assays were performed by culturing the appropriate *E. coli* or *B. cenocepacia* strains overnight in LB and inoculating NG agar with approximately 100 µl of cultures adjusted to 1×10^4^ CFU/ml on 2.0-cm diameter plates. Plates were incubated overnight at 37°C and allowed to cool to before transferring at least 25 L4 larvae or adult worms per plate and incubating plates at 25°C. Using a stereomicroscope (Fischer) at 50× magnification, nematodes were enumerated as alive or dead by gentle prodding with a platinum wire and assessing responsiveness.

### Statistical analyses

All statistical analyses were performed using GraphPad Prism software (GraphPad Software, San Diego, California). For AHL activity assays, motility assays, protease activity assays or promoter::*lux* fusions, all data were analyzed by two-way analysis of variance (ANOVA) and all values are the means ± SD of results from triplicate cultures and are representative of results from at least two individual trials. For motility and protease activity assays, values were recorded at several timepoints and values at one representative timepoint are shown. For rat infections, statistical analysis was performed using an unpaired t test with Welch's correction. For nematode infections, statistical analysis was performed using Kaplan-Meier survival kinetics and the log-rank (Mantel-Cox) test.
